# Perceived healthy community environment and loneliness among older adults in China: indirect psychosocial pathways through community belongingness and multi-source received social support

**DOI:** 10.3389/fpubh.2026.1940959

**Published:** 2026-09-18

**Authors:** Sheng Sun, Yiwen Wang, Dongdong Wang

**Affiliations:** Department of Sociology, School of Law, Jiangnan University, Wuxi, China

**Keywords:** community belongingness, healthy community environment, loneliness, older adults, social support

## Abstract

**Introduction:**

Loneliness in later life is closely linked to older adults' living environments and psychosocial resources. Although digital inclusion has received increasing attention in aging societies, less is known about whether perceived healthy community environments remain relevant to loneliness among older adults. This study examined whether community belongingness (CB) and multi-source received social support (MRSS) formed indirect psychosocial pathways linking perceived healthy community environment (PHCE) to loneliness among older adults in China.

**Methods:**

Data were drawn from a face-to-face cross-sectional survey conducted across 21 communities in Wuxi, China. The analytical sample included 469 adults aged 60 years or older. A serial multiple mediator model was estimated using 5,000 bootstrap samples to assess indirect associations. Models adjusted for demographic, socioeconomic, health-related, and technology-related covariates.

**Results:**

Higher PHCE was associated with stronger CB and higher MRSS. CB and MRSS were both negatively associated with loneliness, whereas the direct association between PHCE and loneliness was not statistically significant after these psychosocial variables were included. Bootstrap analyses indicated statistically significant indirect associations through CB [effect = −0.085, 95% CI (-0.197,−0.004)], through MRSS [effect = −0.046, 95% CI (-0.125,−0.010)], and through the sequential PHCE–CB–MRSS–loneliness pathway [effect = −0.027, 95% CI (-0.082,−0.006)]. Sensitivity analyses showed that MRSS-related pathways were attenuated after adjustment for e-health literacy, while the pathway through CB remained more stable.

**Discussion:**

Perceived healthy community environments may be linked to lower loneliness among older adults through psychosocial pathways involving belongingness and received social support. These findings suggest that community-based loneliness prevention should attend not only to physical and service environments, but also to older adults' psychological attachment and accessible support resources.

## Introduction

The 21st century is marked by two forces reshaping societies worldwide: rapid population aging and pervasive digital transformation. This intersection is particularly pronounced in China. By 2024, China's population aged 60 years and older had reached 310 million. The country had formally entered a moderately aging society, prompting a national “Active Aging” policy focused on health and social participation among older adults (1). A central paradox has emerged. Digital infrastructures designed to enhance connectivity may also contribute to technological alienation among older adults, understood here as feelings of powerlessness, exclusion, or disconnection when digital systems demand competencies they may not possess (2). In this sense, technological alienation refers to an experiential response to digital demands, rather than simply limited access to technology, restricted digital participation, or low digital literacy. Such experiences may be relevant to social engagement and loneliness in later life, although these associations may vary across forms of digital participation. In this context, older adults are not only a vulnerable group but also a theoretically important population for examining the enduring role of physical communities in an increasingly placeless digital era. Although China provides the empirical context for this study, the underlying question is relevant more broadly to aging societies undergoing rapid digital transformation.

The Ecological Perspective on Aging provides a useful framework for interpreting this tension, emphasizing that wellbeing depends on the fit between individual competence and environmental demands (3). From this perspective, digital environments that exceed older adults' competencies may constitute a form of environmental press and contribute to person–environment mismatch, which may be relevant to loneliness in later life (4–6).

Current interventions primarily seek to enhance older adults' digital literacy, improve digital inclusion, and reduce loneliness risks associated with the digital divide. Although these efforts are important, they may understate the continuing relevance of the local community as a proximal social and physical environment. Accordingly, the present study treats digitalization as the broader social context and focuses on whether perceived community environments are associated with loneliness through psychosocial resources. Technology-related characteristics are examined only in supplementary sensitivity analyses. Nevertheless, the psychosocial pathways through which the “power of place” is associated with loneliness remain insufficiently specified.

Three unresolved issues remain in this literature. First, research on community environments and research on digital technology and loneliness have largely developed in separate lines: some studies focus on the health implications of community environments (7, 8) whereas others examine digital technology and loneliness (5, 6, 9). This separation leaves the continuing relevance of place-based community resources in a digitalizing society insufficiently clarified. Second, although the link between community and wellbeing is well established (7, 10, 11), the psychosocial resources accompanying this association remain less clearly specified. Third, community belongingness and received social support are often examined separately, making it difficult to determine whether these affective and support-related resources represent distinct yet interconnected pathways linking perceived community environments to loneliness. Addressing these gaps requires an integrated framework that connects perceived environmental conditions with both affective attachment and received social support.

Against this background, this study examines the relationship between perceived healthy community environment (PHCE) and loneliness among older adults within a digitalizing social context. PHCE refers to residents' subjective assessment of the extent to which their community supports health and healthy daily living and encompasses five domains: physical environment quality, walkability and traffic environment, accessibility of health resources, social activities and services, and community safety and security. Focusing on perceived conditions is consistent with the person–environment perspective adopted in this study, while PHCE should not be interpreted as an objective audit of the built environment. The study asks through what distinct yet interconnected psychosocial pathways PHCE is associated with loneliness. Drawing on the Theory of Sense of Community (12) and Social Capital Theory (13), this study examines indirect associations through community belongingness (CB), through multi-source received social support (MRSS), and through a theoretically specified sequential pathway linking CB and MRSS.

## Literature review and hypotheses

### Theoretical lens: the ecological perspective on aging

The overarching framework of this study, the Ecological Perspective on Aging, explains why community is central to the analysis. Its central tenet, the competence-press model, posits that wellbeing is optimized when individual capabilities are aligned with environmental demands (3). This study conceptualizes technological alienation as a form of environmental press that may exceed older adults' competence and heighten loneliness. This perspective reframes the problem as an environmental mismatch rather than a personal failing among older adults. Consequently, it directs attention away from changing individuals alone, such as through digital training, and toward improving their most immediate environment: the community. Such improvement may create a more supportive context and strengthen person-environment fit.

### The affective pathway of community belongingness

To explain how communities may provide psychological support, this study draws on the Theory of Sense of Community (12). The theory defines sense of community as an affective construct encompassing membership, influence, need fulfillment, and shared emotional connection. This framework helps explain how physical environments may be translated into affective experiences. In this regard, healthy community design, characterized by walkable streets, accessible public spaces, and safe settings, is not merely an aesthetic preference but a social-psychological resource. It may facilitate repeated positive interactions, which form the basis of belongingness (14). Empirical evidence supports this association: community-level factors are associated with sense of community, which is negatively correlated with loneliness. Among older adults, the quality of local public open spaces, rather than proximity alone, was linked to stronger community belongingness (15). Built environment characteristics have also been linked to social outcomes among older adults, including community belongingness and social participation (16). These findings suggest that well-designed environmental features may support this affective state. A stronger sense of belongingness may address a central feature of loneliness: the subjective experience of disconnection (17). Therefore, we hypothesize:

H1: Perceived healthy community environment (PHCE) is indirectly associated with loneliness through community belongingness (CB).

### The structural pathway of multi-source received social support

To explain how communities may provide tangible social resources, this study employs Social Capital Theory (13). The theory views social networks as valuable capital that provides resources such as trust and support. A well-designed community with accessible third places may facilitate the formation of social capital (18). By creating opportunities for interaction, such communities may support both bonding ties and bridging ties. These ties may increase multi-source received social support, which can provide practical resources when older adults face exclusion related to technological alienation. Classic studies have shown the importance of social network diversity for wellbeing (19). Empirical studies also indicate that environments shape social networks. Greater availability of community facilities and improved accessibility are associated with older adults' capacity to sustain broader and more diverse social networks over time (20). Social infrastructure, including parks, libraries, and cafes, has been associated with lower loneliness and greater social network diversity (21, 22). These places may create everyday opportunities for interpersonal connection. Thus, the community's role extends beyond fostering affective attachment to supporting tangible social networks. We therefore propose:

H2: PHCE is indirectly associated with loneliness through multi-source received social support (MRSS).

### The sequential affect-to-structure pathway

This study integrates two mid-range theories to propose a sequential association between affective and structural psychosocial resources. Community belongingness represents an affective connection to place, whereas MRSS reflects support received across multiple social and institutional sources. Theoretically, stronger belongingness may be associated with greater willingness to participate and interact, consistent with evidence that affective states can motivate prosocial behavior relevant to social capital formation (23). Several empirical studies have also linked community belongingness with social engagement and related mental-health outcomes (10, 16, 24). On this basis, CB is specified before MRSS in the serial model. This ordering reflects the theoretical structure of the proposed model rather than empirically established temporal precedence, which cannot be determined from the present cross-sectional data. This leads to the final hypothesis:

H3: PHCE is indirectly associated with lower loneliness through a sequential pathway involving higher CB and higher MRSS.

## Methods

### Participants

Data for this cross-sectional study were drawn from the “Community Residents' Healthy Living Status and Healthy Community Construction Project”, a large-scale university–community partnership initiative conducted from January to May 2025 across 21 communities in Wuxi City, Jiangsu Province, China. The parent study used non-probability quota sampling to recruit adults aged 18 years and older. Quota controls were implemented separately within each community and were defined by age (18–35 years, 36–59 years, and ≥60 years) and gender, yielding six demographic cells. Target numbers for each cell were set with reference to the population structure of the respective community. Within communities, participants were recruited through fixed-location intercept recruitment with assistance from staff at community civil-affairs service stations; recruitment for a given cell stopped once its target had been reached. Eligibility criteria included prior experience with digital health products or services and the ability to complete the questionnaire independently, while individuals with severe cognitive or physical impairments that prevented independent responses were excluded. Questionnaires were completed face to face in the presence of trained fieldworkers, who could provide procedural guidance or clarify item wording when requested but were instructed not to suggest or influence responses.

A total of 2,200 questionnaires were distributed, and 2,130 were returned. During data coding, questionnaires showing extensive straight-line or patterned responding across substantial portions of the instrument, or substantial incompleteness, were flagged and manually reviewed. The two quality-control criteria could overlap and were not treated as mutually exclusive. After quality screening, 1,874 valid records remained. The present study then restricted the dataset to adults aged 60 years or older: 1,405 respondents were younger than 60 years and were outside the target population, leaving 469 older adults for the present analysis. Because prior experience with digital health products or services was an eligibility criterion of the parent survey, the older-adult subsample represents individuals with at least some prior digital-health exposure. An *a priori* power analysis was conducted in G^*^Power 3.1 using the fixed-model linear multiple regression R^2^-increase procedure (f^2^ = 0.15, α = 0.05, power = 0.95, two tested predictors, 11 total predictors), yielding a minimum required sample size of 169.

Detailed demographic characteristics of the sample are presented in Table 1. The mean age was 65.3 years (SD = 4.46), and the gender distribution was nearly balanced (53.7% male). In socioeconomic terms, the sample had moderate levels of formal education; 38.4% had completed primary school or less. A majority of participants (54.4%) reported a monthly income below 5,000 Chinese yuan (CNY). Before retirement, many had worked in self-employment, agriculture, or the private sector. Only 4.1% of respondents lived alone. Participants reported a mean self-rated health score of 4.01 (SD = 0.828), a mean technology readiness score of 3.56 (SD = 0.461), and a mean e-health literacy score of 4.23 (SD = 0.641).

**Table 1:** Demographic characteristics of participants (*N* = 469).

Characteristics	Frequency/ Mean	Percentage/ S.D.
Age (years)	65.3	4.46
Gender		
Male	252	53.7
Female	217	46.3
Educational level		
Primary school or below	180	38.4
Middle school	138	29.4
High school	106	22.6
College or above	44	9.6
Monthly income (CNY)		
Less than 5,000	255	54.4
5,000–7,000	111	23.7
More than 7,000	103	21.9
Previous occupation (before retirement)		
Public sector (government, institution, state-owned enterprise)	16	3.4
Private sector enterprise	153	32.6
Self-employed/Agricultural worker	233	49.7
Unemployed	66	14.1
Number of cohabiting family members		
0 (Living alone)	19	4.1
1	241	51.6
2 or more	208	44.3
Marital status		
Not currently married	73	15.6
Married (with spouse present)	396	84.4
Self-rated health	4.01	0.828
Technology readiness	3.56	0.461
E–health literacy	4.23	0.641

### Measurement

The measures included two categories: Chinese translations of validated scales, including community belongingness and technology readiness, and an adapted scale derived from multiple international instruments and tailored to the Chinese context, namely perceived healthy community environment. For the Chinese translations, a standardized protocol was followed, including forward translation, back-translation, consistency checks, and bias correction. Native English speakers and subject-matter experts jointly reviewed the process to support linguistic rigor and conceptual accuracy. For the adapted scale, the item pool was developed by synthesizing core items from relevant international instruments. It was then refined through literature review, expert evaluation, and cognitive interviews with the target population to fit the Chinese sociocultural context. The reliability and validity of the scales were first assessed in a pilot study (*n* = 154) and then examined in the main survey. Overall, the measures showed acceptable psychometric properties for social science research.

### Dependent variable

Loneliness was measured using the Chinese version of the Short-Form UCLA Loneliness Scale (ULS-8), adapted by Li (25) from the original UCLA Loneliness Scale developed by Russell, Peplau (26). Participants rated the feelings described in each item, such as “I often feel a lack of companions.” Responses were recorded on a 6-point Likert scale ranging from 1 (completely disagree) to 6 (completely agree). After reverse-scoring the relevant items, a mean score was calculated. Higher scores indicated greater loneliness. The scale showed acceptable internal consistency in the current study (KMO = 0.851, *p* < 0.001; Cronbach's α = 0.791).

### Independent Variable

Perceived healthy community environment (PHCE) was defined as residents' subjective perception of the extent to which their neighborhood environment supports health and healthy lifestyles. Because no widely accepted scale fully captured the context of comprehensive urban communities in China, the PHCE measure was developed by drawing on established theoretical frameworks and empirical instruments. Its conceptual dimensions were informed by the WHO Age-Friendly Cities framework and broader healthy-cities planning principles (27), while specific built-environment items were adapted from internationally recognized instruments, including the Neighborhood Environment Walkability Scale (NEWS), the Physical Activity Neighborhood Environment Scale (PANES), and related short-form versions (28–31).

The final 13-item PHCE measure retained five core domains: physical environment quality, walkability and traffic environment, accessibility of health resources, social activities and services, and community safety and security. All items were rated on a 6-point Likert scale (1 = completely disagree, 6 = completely agree), with negatively worded items reverse-scored before calculating the mean. Higher scores represented a more supportive perceived community environment. Standardized factor loadings for the retained items are reported in Supplementary Table S1. The measure showed acceptable internal consistency and composite reliability (Cronbach's α = 0.757; CR = 0.928; KMO = 0.792; Bartlett's test, *p* < 0.001). Its AVE was 0.420, below the conventional 0.50 benchmark, and convergent validity was therefore interpreted cautiously. The retained item set nevertheless preserved representation of all five content domains, and HTMT was 0.723.

### Mediating variables

Community belongingness (CB; M1) was assessed using the 8-item Brief Sense of Community Scale (32). Participants responded to items such as “I feel that I am a part of the community” on a 6-point Likert scale (1 = completely disagree, 6 = completely agree). A mean score was calculated, with higher scores reflecting stronger community belongingness. The scale showed acceptable reliability in the current sample (KMO = 0.814, *p* < 0.001; Cronbach's α = 0.718).

Multi-source received social support (MRSS) is conceptualized as a composite indicator that reflects individuals overall received social support across various sources and dimensions. The MRSS indicators represent distinct components of received support that collectively contribute to the overall support profile. The MRSS measure comprises 21 indicators assessing the frequency with which participants received emotional, informational, and practical support from seven distinct sources. These sources include family or relatives, friends or colleagues, community neighbors, workplace contacts, social organizations or professional services (e.g., medical staff and legal aid providers), government agencies, and online platforms (e.g., online friends and internet forums). Participants rated the frequency of support from each source on a 6-point Likert scale (0 = never, 5 = always). Each indicator was equally weighted, and the final MRSS score was calculated as the mean value of all 21 indicators. Higher scores indicate greater received social support from multiple sources and across different types of support. The detailed structure and scoring procedure of the MRSS are presented in Supplementary Table S2.

Discriminant validity among the key study constructs was additionally examined using the heterotrait–monotrait ratio (HTMT). The HTMT statistics are reported in Supplementary Table S3. Because MRSS was operationalized as a multi-component composite indicator rather than a reflective latent construct, traditional reflective measurement criteria were not applied to MRSS.

### Covariates

Demographic and technology-related variables were included as covariates to account for potential confounding. Demographic covariates included age (continuous), gender (0 = male, 1 = female), education level (categorical: 1 = primary school or below, 4 = college or above), monthly income (categorical: 1 = less than 5,000 CNY, 4 = more than 7,000 CNY), pre-retirement employment status (1 = employed, 0 = unemployed), number of cohabiting family members (categorical: 1 = 0, 3 = 2 or more), marital status (1 = not currently married, 2 = married), and self-rated health (1 = very poor, 5 = very good).

Two technology-related variables were also included. Technology readiness was assessed using Parasuraman's (2000) 12-item Technology Readiness Index (TRI), which covers four dimensions: optimism, innovativeness, discomfort, and insecurity. Items were rated on a 6-point Likert scale, and the scale showed acceptable reliability (KMO = 0.822, *p* < 0.001; Cronbach's α = 0.766). E-health literacy was measured using the 15-item Digital Health Literacy Assessment Scale (Liu, 2022), which includes two dimensions: access/assessment and interaction/application. Items were rated on a 6-point Likert scale, and the scale showed good reliability (KMO = 0.886, *p* < 0.001; Cronbach's α = 0.840). These technology-related variables were used to examine whether the community-based pathways were sensitive to individual technological orientations and e-health literacy.

### Statistical analysis

All analyses were conducted using SPSS 29.0 and PROCESS 3.5. Preliminary analyses included descriptive statistics and Pearson correlations for the key variables. Internal consistency of multi-item reflective scales was assessed using Cronbach's alpha. No missing-value imputation was performed; after questionnaire-level quality screening, each model was estimated using complete cases for the variables included in that analysis. To test the hypothesized indirect associations, PROCESS Model 6 was used to estimate a serial multiple mediator model with PHCE as X, CB as M1, MRSS as M2, and loneliness as Y (33). Indirect associations through CB, through MRSS, and through the sequential CB–MRSS pathway were evaluated using 5,000 bootstrap samples and 95% bias-corrected confidence intervals; an indirect association was considered statistically significant when its confidence interval did not include zero.

The primary models adjusted for age, gender, educational attainment, monthly income, pre-retirement employment status, number of cohabiting family members, marital status, and self-rated health. Two supplementary sensitivity models were then estimated separately, one additionally adjusting for technology readiness and the other for e-health literacy. Harman's single-factor test was used as a diagnostic check for potential common method bias. PROCESS Model 6 was used to estimate theoretically specified indirect associations.

## Results

### Participant characteristics and preliminary analyses

Descriptive statistics and Pearson correlation coefficients for the key study variables are presented in Table 2. Participants reported moderate-to-high levels of PHCE (M = 4.28, SD = 0.446) and CB (M = 4.35, SD = 0.603). The mean loneliness score was relatively low (M = 3.46, SD = 0.662), whereas MRSS was moderate (M = 3.20, SD = 0.627). Preliminary correlations were in the expected directions. The correlation matrix indicated that loneliness was negatively associated with PHCE (*r* = −0.112, *p* < 0.01), CB (*r* = −0.139, *p* < 0.01), and MRSS (*r* = −0.145, *p* < 0.01). PHCE was positively associated with both CB (*r* = 0.539, *p* < 0.01) and MRSS (*r* = 0.466, *p* < 0.01).

**Table 2:** Descriptive statistics and correlations among main variables.

Variable	Mean	S.D.	LON	PHCE	CB	MRSS
Loneliness (LON)	3.46	0.662	1			
Perceived healthy community environment (PHCE)	4.28	0.446	−0.112^**^	1		
Community belongingness (CB)	4.35	0.603	−0.139^**^	0.539^**^	1	
Multi-source received social support (MRSS)	3.20	0.627	−0.145^**^	0.466^**^	0.483^**^	1

^**^p < 0.01.

Harman's single-factor test was used to assess potential common method bias. An unrotated exploratory factor analysis including all scale items identified 12 factors with eigenvalues greater than 1. The first factor accounted for 16.05% of the variance, below the 40% threshold, suggesting no serious common method bias in the sample. Nevertheless, given the limited sensitivity of Harman's single-factor test, this finding should be interpreted cautiously. Multicollinearity diagnostics showed that tolerance values were above 0.1 (range: 0.372–0.935) and variance inflation factor (VIF) values were approximately below 3 (range: 1.069–3.015), indicating no serious multicollinearity (34). These preliminary findings were consistent with the pathways proposed in the mediation model.

### Serial indirect-effect analysis

To test the hypothesized indirect pathways linking perceived community environment and loneliness, this study used the PROCESS macro for SPSS (Model 6) to estimate a serial multiple mediator model with 5,000 bootstrap resamples and 95% bias-corrected confidence intervals. Demographic and health-related variables were included as covariates. Full results are presented in Table 3, and path coefficients are visualized in Figure 1.

**Table 3:** Results of the serial multiple mediator model linking perceived healthy community environment to loneliness.

Variables	Model 1 (LON)		Model 2 (CB)		Model 3 (MRSS)		Model 4 (LON)	
	B	t	B	t	B	t	B	t
Intercept	3.938	7.297^***^	1.651	3.744^***^	−0.007	−0.016	4.208	7.779^***^
PHCE	−0.119	1.996^*^	0.704	12.794^***^	0.371	5.710^***^	0.039	0.501
CB					0.314	6.623^***^	−0.121	−2.096^*^
MRSS							−0.124	−2.285^*^
AGE	0.024	3.726^***^	−0.013	−2.299^**^	−0.014	−2.525^**^	0.021	3.149^**^
SEX	0.071	1.215	0.031	0.627	0.048	0.961	0.082	1.417
EDU	−0.099	−1.972^*^	−0.055	−1.301	−0.004	−0.096	−0.108	−2.170^*^
MAI	−0.120	−3.765^***^	0.016	0.595	0.134	4.936^***^	−0.139	−4.306^***^
PES	−0.074	−1. 676	−0.018	−0.476	0.059	1.568	−0.070	−1.589
NCFM	0.021	0.590	−0.034	−1.156	−0.062	−1.208	0.014	0.418
MS	−0.090	−1.189	0.137	2.153^*^	0.115	1.776	−0.054	−0.715
SRH	−0.330	−7.720^***^	0.074	2.072^*^	0.122	3.328^***^	−0.302	−7.060^***^
R^2^	0.191		0.311		0.349		0.215	
F	12.020^***^		22.930^***^		24.428^***^		11.326^***^	

^*^p < 0.05, ^**^p < 0.01, ^***^p < 0.001.

LON, loneliness; PHCE, perceived healthy community environment; CB, community belongingness; MRSS, multi-source received social support; AGE, age; SEX, sex; EDU, educational attainment; MAI, monthly average income; PES, pre-retirement employment status; NCFM, number of cohabiting family members; MS, marital status; SRH, self-rated health.

Reference categories: male, unemployed, married, and local residents.

**Figure 1:**
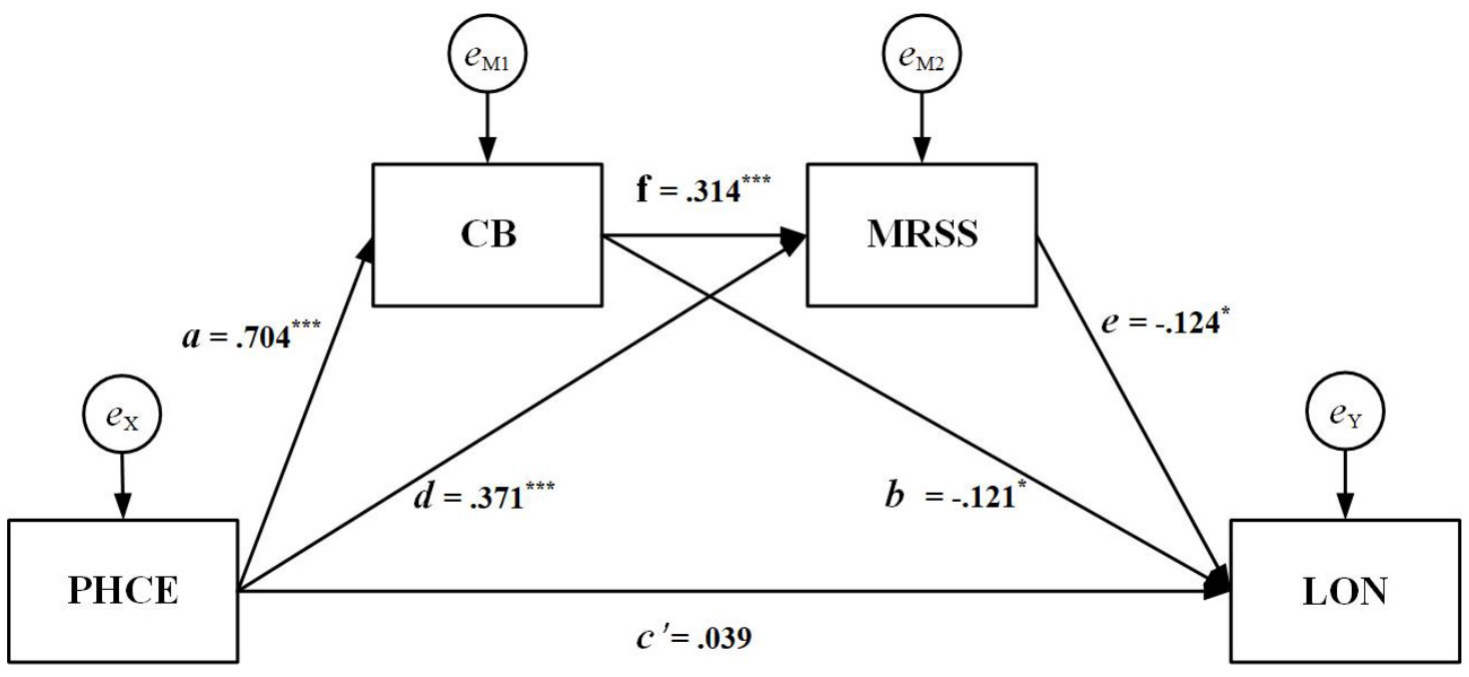
Path Coefficients of the Serial Multiple Mediator Model. ^*^p < 0.05 ^**^p < 0.01 ^***^p < 0.001. LON = Loneliness; PHCE = Perceived Healthy Community Environment; CB = Community Belongingness; MRSS = Multi-source Received Social Support.

The serial multiple mediator model produced results consistent with the proposed community-based pathways. Higher PHCE was associated with greater CB (B = 0.704, *p* < 0.001) and greater MRSS (B = 0.371, *p* < 0.001). Community belongingness was positively associated with MRSS (B = 0.314, *p* < 0.001), supporting the hypothesized serial link between the two mediators. To further examine the incremental contribution of community belongingness to received social support, a hierarchical regression analysis was conducted. After entering demographic and socioeconomic covariates, the addition of community belongingness significantly increased the explained variance of MRSS from R^2^ = 0.176 to R^2^ = 0.349 (ΔR^2^ = 0.173, *p* < 0.001). In the final model predicting loneliness, both CB (B = −0.121, *p* < 0.05) and MRSS (B = −0.124, *p* < 0.05) were negative predictors. After accounting for CB and MRSS, the direct association between PHCE and loneliness was not statistically significant (B = 0.039, *p* = 0.501), suggesting that the PHCE-loneliness association was primarily indirect in this model.

The bootstrap analysis showed the indirect effects reported in Table 4. The indirect association between PHCE and loneliness through CB was statistically significant [effect = −0.085, 95% CI (-0.197,−0.004)], consistent with Hypothesis 1. The indirect association through MRSS was also statistically significant [effect = −0.046, 95% CI (-0.125,−0.010)], supporting Hypothesis 2. The sequential indirect pathway involving CB and MRSS was also statistically significant [effect = −0.027, 95% CI (-0.082,−0.006)], consistent with Hypothesis 3. Collectively, these findings were consistent with the proposed model. They suggest an associational pattern in which a favorable community environment is linked to lower loneliness alongside stronger community belongingness and broader social support networks, including a sequential pathway in which belongingness is associated with broader support.

**Table 4:** Bootstrap tests of serial indirect effects.

Paths	Total effect	Direct effect	Indirect effect	95% CI	
				Lower	Upper
PHCE → LON	−0.119	0.039	−0.158	−0.069	0.252
PHCE → CB → LON	−0.085	0.000	−0.085	−0.197	−0.004
PHCE → MRSS → LON	−0.046	0.000	−0.046	−0.125	−0.010
PHCE → CB → MRSS → LON	−0.027	0.000	−0.027	−0.082	−0.006
PHCE → CB	0.703	0.703	0.000	0.584	0.819
PHCE → MRSS	0.359	0.359	0.000	0.220	0.501
CB → MRSS	0.329	0.329	0.000	0.193	0.452
CB → LON	−0.139	−0.139	0.000	−0.280	−0.005
MRSS → LON	−0.183	−0.183	0.000	−0.313	−0.029

LON, loneliness; PHCE, perceived healthy community environment; CB, community belongingness; MRSS, multi-source received social support.

### Robustness and diagnostic checks

To assess robustness and account for the potential influence of individual-level technological capacities, this study conducted two supplementary sensitivity analyses. First, the model was re-estimated with technology readiness included as a covariate. As shown in Supplementary Table S4, the results were substantively unchanged from the primary model. The core path coefficients retained their direction and statistical significance. Crucially, all three indirect effects remained statistically significant, suggesting that the model was robust to adjustment for general attitudes toward technology. The indirect effects were as follows: via CB, effect = −0.092, 95% CI [-0.167,−0.009]; via MRSS, effect = −0.065, 95% CI [-0.119,−0.015]; and via CB and MRSS, effect = −0.048, 95% CI [-0.089,−0.011]. Second, an additional model was estimated with e-health literacy included as a covariate. This analysis qualified the primary findings (see Supplementary Table S5). Although the PHCE-CB path remained statistically significant (B = 0.611, *p* < 0.001), the MRSS-loneliness path was no longer statistically significant (B = −0.063, *p* = 0.071).

This change affected the observed indirect pathways. Specifically, the indirect effect via CB alone remained statistically significant [effect = −0.063, 95% CI (-0.174,−0.011)], whereas the two MRSS-related pathways, the parallel path (H2) and the sequential indirect path (H3), were no longer statistically significant. This finding suggests that the apparent loneliness-buffering role of diverse support networks may depend on e-health literacy. In other words, after statistically adjusting for individuals' capacity to access and use online resources, the association between MRSS and loneliness was attenuated. By contrast, the CB pathway remained significant, suggesting a more stable place-based relational association.

## Discussion

This study examined the association between perceived healthy community environment and loneliness among older adults in the broader context of digitalization in later life. The empirical analysis focused on community-based psychosocial pathways, while technological alienation served as a contextual concept rather than a directly measured construct. The findings suggest that community belongingness and multi-source received social support are relational correlates within indirect pathways linking perceived community environments and loneliness. Four main points emerge from the findings.

First, the analysis points to the indirect relevance of perceived community environments. The direct association between PHCE and loneliness was not statistically significant after CB and MRSS were included, whereas the indirect associations were supported. Thus, a supportive perceived community environment should not be interpreted as a direct antidote to loneliness; rather, its relevance appears to lie in its association with relational resources such as community belongingness and received social support. This interpretation is consistent with the Ecological Perspective on Aging (3), which emphasizes transactions between individuals and their environments, and extends prior work focusing on direct associations between physical environments and older adults' health (8) or on social participation (35). The coefficient pattern also showed a sign reversal: the total association was negative, whereas the direct coefficient became small and positive after inclusion of CB and MRSS (B = 0.039, *p* = 0.501), while the combined indirect association remained negative. This pattern is compatible with inconsistent mediation or a suppression-like pattern, but the nonsignificant direct coefficient and cross-sectional design do not support interpreting it as a distinct causal suppression mechanism.

Second, the results highlight community belongingness as an affective correlate within the proposed pathway. PHCE was positively associated with CB, and CB was in turn positively associated with MRSS. The observed sequential indirect association is therefore consistent with an affect-to-structure interpretation, but the ordering should be understood as theoretically specified rather than temporally demonstrated. Previous studies have linked community belongingness with social engagement and have shown that social interaction and group-based relationships are relevant to older adults' wellbeing and loneliness (36, 37). Taken together, the present findings suggest that affective attachment to the community and broader received-support networks are closely connected relational resources. This study therefore shifts attention from the provision of external opportunities alone to the affective conditions that may enable older adults to benefit from those opportunities.

Third, the sensitivity analyses suggest that the MRSS-related associations were more sensitive to e-health literacy than the CB pathway. When e-health literacy was added as a covariate, the two MRSS-related indirect pathways were no longer statistically significant, whereas the indirect association through CB remained statistically significant. This attenuation suggests that the association between broader received support and loneliness may partly overlap with individual differences in the capacity to access and use digital resources. One possible interpretation is that older adults with greater e-health literacy have additional online channels through which support can be obtained, which may reduce the distinct contribution of place-based support networks. This interpretation is consistent with prior work suggesting that digital ties can complement offline networks (38). Because e-health literacy entered the model as a covariate rather than as a tested moderator, the present results support viewing digital and place-based resources as potentially complementary rather than as demonstrated substitutes.

Finally, the comparison of the two sensitivity analyses suggests that different technology-related characteristics may not be interchangeable. Adjustment for technology readiness left the main pattern largely unchanged, whereas adjustment for e-health literacy attenuated the MRSS-related pathways. This difference suggests that more specific digital skills may account for variation in support-related associations beyond general attitudes toward technology. Because these analyses were supplementary and observational, however, the finding should be interpreted as exploratory rather than as evidence that digital competence changes older adults' reliance on physical communities.

### Practical implications

These findings offer practical guidance for developing age-friendly communities in the digital age. First, community programs should activate physical infrastructure rather than focus on infrastructure provision alone. Within the dimensions captured by PHCE, relevant improvements may include barrier-free and continuous walking routes, adequate lighting, safer traffic environments, accessible health resources, and usable shared spaces for recurring community activities. Such physical improvements can be combined with programs that make these spaces socially meaningful, including cultural activities and mutual-aid initiatives that create regular opportunities for older residents to meet and interact (7).

Second, communities should strengthen belonging-oriented practices. Rather than relying mainly on one-off social events, such practices can emphasize continuity, role recognition, and resident participation. Examples include stable interest-based small groups, peer-volunteer roles, resident co-design of community activities, neighborhood mutual-aid arrangements, and mechanisms for older adults to participate in community governance. These approaches are consistent with the broader aim of supporting sustained social connection, as illustrated by programs such as the UK's Befriending schemes (39).

Third, place-based and digital forms of support should be integrated. Age-friendly community development can combine environmental improvement, belonging-oriented activities, and accessible support channels. Existing place-based approaches such as community gardens, walking groups, mutual-aid programs, and resident participation mechanisms can be complemented by face-to-face service desks, peer digital assistance, and support for navigating online health or public-service systems. At the same time, offline service options should remain available for older adults who prefer or require them. In this way, digital tools can improve information access and service coordination without replacing opportunities for everyday face-to-face interaction. These recommendations should be read as applications consistent with the observed associations, rather than as interventions whose effectiveness has been demonstrated by the present study.

### Limitations and future research

Several limitations should be acknowledged. First, the cross-sectional design precludes causal inference and does not establish the temporal ordering of PHCE, CB, MRSS, and loneliness. The indirect pathways should therefore be interpreted as theoretically informed associational patterns; alternative orderings, including a pathway in which received support is associated with subsequent or concurrent belongingness, are also compatible with the data. Second, all focal variables were self-reported. PHCE captures individual perceptions of community conditions rather than objectively measured or community-level environmental characteristics, and the use of self-reported PHCE together with CB, MRSS, and loneliness may increase shared method variance. Harman's single-factor test was used only as a diagnostic check and cannot rule out common method bias. Third, the study used non-probability quota sampling in Wuxi, and eligibility for the parent survey required prior experience with digital health products or services. The findings therefore should not be generalized to the wider older population, particularly those with no digital experience or the most severe digital exclusion. Fourth, although the PHCE measure showed acceptable internal consistency and composite reliability, its AVE was below the conventional benchmark, indicating limited convergent validity; further validation in broader samples is warranted. Fifth, MRSS was operationalized as an equally weighted composite across multiple support sources and types. This approach captures an overall support profile but may obscure heterogeneity between particular sources or forms of support, which future studies could examine separately.

## Conclusion

In an era shaped by population aging and digital transformation, this study suggests that place-based community resources remain relevant to older adults' loneliness. By integrating perceived healthy community environment, community belongingness, and multi-source received social support within a single theoretically specified model, the study extends prior work beyond a simple direct environment–loneliness association. The observed indirect associations suggest that the relevance of perceived community environments may be closely tied to relational resources involving both affective attachment and received support. For practice, age-friendly community development may therefore benefit from combining accessible and safe local environments with sustained opportunities for belonging, mutual assistance, and support access, while using digital tools as a complement rather than a replacement for place-based interaction. Because the evidence is cross-sectional and PHCE is an individual-level perception, future longitudinal and multilevel studies are needed to determine whether these pathways unfold over time and whether they generalize to older adults with little or no digital experience.

## Data Availability

The datasets presented in this article are not readily available because their containing information that could compromise the privacy of research participants. The data that support the findings of this study are available on request from the corresponding author. Requests to access the datasets should be directed to Dongdong Wang, Dongdongwang@jiangnan.edu.cn.
