# Supplementary material for: Perceived healthy community environment and loneliness among older adults in China: indirect psychosocial pathways through community belongingness and multi-source received social support
Source: Front Public Health. 2026 Sep 18;14:1940959. doi: 10.3389/fpubh.2026.1940959 (PMC13630697; doi:10.3389/fpubh.2026.1940959)
Supplement: Supplementary file 1 [file Table_1.docx]

Table S1. Detailed Items and Psychometric Properties of the PHCE Scale

| Construct  (Variable) | Dimensions | Questionnaire items | Loading | α | CR | AVE | KMO | Bartlett | HTMT  (Max) |
| --- | --- | --- | --- | --- | --- | --- | --- | --- | --- |
| Perceived healthy community environment (PHCE) | Physical Environment Quality | Air quality in the community is good. | 0.633 | .757 | .928 | .420 | .792 | P < .000 | .723 |
|  |  | There is noise pollution in the community. (R) | 0.646 |  |  |  |  |  |  |
|  |  | The community has insufficient green spaces. (R) | 0.628 |  |  |  |  |  |  |
|  | Walkability and Traffic Environment | There are continuous walkways available for residents to use in the community. | 0.662 |  |  |  |  |  |  |
|  |  | The community has moderate traffic flow and high traffic safety. | 0.686 |  |  |  |  |  |  |
|  | Accessibility of Health Resources | The accessibility of community medical services is poor. (R) | 0.62 |  |  |  |  |  |  |
|  |  | The community is equipped with sports facilities or rehabilitation centers. | 0.699 |  |  |  |  |  |  |
|  |  | The community provides health education and training (e.g., healthy diet, chronic disease management, etc.). | 0.653 |  |  |  |  |  |  |
|  | Social activities and services | The community often organizes social and cultural activities for residents. | 0.61 |  |  |  |  |  |  |
|  |  | The community frequently offers residents opportunities to participate in volunteer activities. | 0.666 |  |  |  |  |  |  |
|  |  | The community provides social support services to assist residents (e.g., neighborhood mutual assistance, elderly care, psychological support, social activities, etc.). | 0.659 |  |  |  |  |  |  |
|  | Community safety and security | The community has measures to protect residents’ safety, such as security patrols and monitoring equipment. | 0.671 |  |  |  |  |  |  |
|  |  | Community lighting is sufficient to support pedestrian safety at night. | 0.632 |  |  |  |  |  |  |

Note: α = Cronbach’s α; CR = Composite Reliability; AVE = Average Variance Extracted; KMO = Kaiser-Meyer-Olkin Measure of Sampling Adequacy; Bartlett = Bartlett's Test of Sphericity; HTMT (Max) = maximum Heterotrait-Monotrait Ratio.
