# Supplementary material for: Perceived healthy community environment and loneliness among older adults in China: indirect psychosocial pathways through community belongingness and multi-source received social support
Source: Front Public Health. 2026 Sep 18;14:1940959. doi: 10.3389/fpubh.2026.1940959 (PMC13630697; doi:10.3389/fpubh.2026.1940959)
Supplement: Supplementary file 2 [file Table_2.docx]

Table S2. Structure and Operationalization of Multi-source Received Social Support

| Panel A. Structure of MRSS |  |  |  |
| --- | --- | --- | --- |
| Source of support | Emotional support | Informational support | instrumental support |
| Family/relatives | MARSS1 | MARSS2 | MARSS3 |
| Friends/colleagues | MARSS4 | MARSS5 | MARSS6 |
| Community neighbors | MARSS7 | MARSS8 | MARSS9 |
| Work units | MARSS10 | MARSS11 | MARSS12 |
| Social organizations/professional services | MARSS13 | MARSS14 | MARSS15 |
| Government agencies | MARSS16 | MARSS17 | MARSS18 |
| Online platforms | MARSS19 | MARSS20 | MARSS21 |
| Panel B. Composite scoring procedure |  |  |  |
| Component | Description |  |  |
| Number of indicators | 21 indicators | | |
| Structure | 7 support sources × 3 support types | | |
| Weighting method | Equal weighting | | |
| Calculation | MRSS_M = Mean(MARSS1, MARSS2, …, MARSS21) | | |
| Interpretation | Higher scores indicate greater received social support across multiple sources and types | | |
