# Supplementary material for: Perceived healthy community environment and loneliness among older adults in China: indirect psychosocial pathways through community belongingness and multi-source received social support
Source: Front Public Health. 2026 Sep 18;14:1940959. doi: 10.3389/fpubh.2026.1940959 (PMC13630697; doi:10.3389/fpubh.2026.1940959)
Supplement: Supplementary file 3 [file Table_3.docx]

Table S3. Discriminant Validity Among Study Constructs (HTMT)

| Construct | PHCE | CB | MRSS |
| --- | --- | --- | --- |
| PHCE | - |  |  |
| CB | 0.723 | - |  |
| MRSS | 0.58 | 0.612 | - |

Note HTMT values represent heterotrait–monotrait ratios. All values were below the recommended threshold of 0.85, indicating satisfactory discriminant validity among the study constructs.
