# Supplementary material for: Perceived healthy community environment and loneliness among older adults in China: indirect psychosocial pathways through community belongingness and multi-source received social support
Source: Front Public Health. 2026 Sep 18;14:1940959. doi: 10.3389/fpubh.2026.1940959 (PMC13630697; doi:10.3389/fpubh.2026.1940959)
Supplement: Supplementary file 4 [file Table_4.docx]

Table S4. Robustness check: indirect-effect analysis with technology readiness as a covariate

| Variables | Model 1  (LON) | | Model 2  (CB) | | Model 3  (MRSS) | | Model 4  (LON) | |
| --- | --- | --- | --- | --- | --- | --- | --- | --- |
|  | B | t | B | t | B | t | B | t |
| Intercept | 2.581 | 4.383^***^ | 1.651 | 3.744^***^ | -.792 | -1.644 | 3.830 | 6.690^***^ |
| PHCE | -.125 | 1.996^*^ | .705 | 12.650^***^ | .328 | 5.126^***^ | .028 | .724 |
| CB |  |  |  |  | .346 | 6.827^***^ | -.112 | -1.985^*^ |
| MRSS |  |  |  |  |  |  | -.149 | -2.678^**^ |
| TR | .086 | 1.393 | -.008 | .883 | .256 | 4.970^***^ | .122 | 1. 974^*^ |
| AGE | .025 | 3.818^***^ | -.012 | 2.301^*^ | -.012 | -2.324^*^ | .021 | 3.243^**^ |
| SEX | .070 | 1.209 | .031 | .627 | .046 | .958 | .082 | 1.430 |
| EDU | -.095 | -1.885 | -.055 | -1.305 | -.007 | -.177 | -.103 | -2.064^*^ |
| MAI | -.116 | -3.632^***^ | .016 | .605 | .122 | 4.607^***^ | -.137 | -4.247^***^ |
| PES | -.080 | -1.789 | -.017 | -.460 | .042 | 1.143 | -.077 | -1.742 |
| NCFM | .021 | .612 | -.034 | -1.157 | -.003 | -.134 | .015 | .446 |
| MS | -.087 | -1.159 | .137 | 2.147^*^ | .122 | 1.928 | -.048 | -.635 |
| SRH | -.333 | -7.789^***^ | 075 | 2.074^*^ | .112 | 3.150^**^ | -.304 | -7.114^***^ |
| R^2^ | .195 | | .311 | | .382 | | .222 | |
| F | 11.034^**^ | | 20.595^***^ | | 25.607^***^ | | 10.769^***^ | |

**p<0.05 **p<0.01 ***p<0.001*

Note: LON = loneliness; PHCE = perceived healthy community environment; CB = community belongingness; MRSS = multi-source received social support; TR = technology readiness; AGE = age; SEX = sex; EDU= educational attainment (years); MAI= monthly average income; PES = Previous employment status; NCFM = Number of cohabiting family members; MS = marital status; SRH = self-rated health.

Reference categories: male, unemployed, married, and local residents.
