# Supplementary material for: Perceived healthy community environment and loneliness among older adults in China: indirect psychosocial pathways through community belongingness and multi-source received social support
Source: Front Public Health. 2026 Sep 18;14:1940959. doi: 10.3389/fpubh.2026.1940959 (PMC13630697; doi:10.3389/fpubh.2026.1940959)
Supplement: Supplementary file 5 [file Table_5.docx]

Table S5. Diagnostic check: indirect-effect analysis with e-health literacy as a covariate

| Variables | Model 1  (LON) | | Model 2  (CB) | | Model 3  (MRSS) | | Model 4  (LON) | |
| --- | --- | --- | --- | --- | --- | --- | --- | --- |
|  | B | t | B | t | B | t | B | t |
| Intercept | 4.340 | 8.089^***^ | 1.395 | 3.081^**^ | -.448 | -1.002 | 4.479 | 8.287^***^ |
| PHCE | .003 | .049 | .611 | 10.373^***^ | .248 | 3.865^***^ | .092 | 1.170 |
| CB |  |  |  |  | .255 | 5.560^***^ | -.104 | -1.987^*^ |
| MRSS |  |  |  |  |  |  | -.063 | -1.105 |
| EHL | -.212 | -4.439^***^ | .159 | 3.957^***^ | .285 | 7.123^***^ | -.175 | -3.435^***^ |
| AGE | .022 | 3.348^***^ | .011 | 1.936 | -.011 | -2.083^*^ | .020 | 3.031^**^ |
| SEX | .089 | 1.551 | .017 | .356 | .025 | .537 | .093 | 1.620 |
| EDU | -.087 | -1.751 | -.064 | -1.540 | -.024 | -.585 | -.096 | -1.939 |
| MAI | -.113 | -3.627^***^ | .021 | .791 | .144 | 5.570^***^ | -.125 | -3.878^***^ |
| PES | -.076 | -1.744 | -.017 | -.454 | .060 | 1.677 | -.074 | -1.704 |
| NCFM | .006 | .175 | -.023 | -.791 | .012 | .403 | .004 | .117 |
| MS | -.075 | -1.011 | .123 | 2.006^*^ | .103 | 1.675 | -.054 | -.720 |
| SRH | -.299 | -7.077^***^ | 052 | -1.454 | .086 | 2.455^*^ | -.288 | -6.772^***^ |
| R^2^ | .225 | | .334 | | .414 | | .234 | |
| F | 13.232^***^ | | 22.865^***^ | | 29.243^***^ | | 11.612^***^ | |

**p<0.05 **p<0.01 ***p<0.001*

Note: LON = loneliness; PHCE = perceived healthy community environment; CB = community belongingness; MRSS = multi-source received social support; TR = technology readiness; AGE = age; SEX = sex; EDU= educational attainment (years); MAI= monthly average income; PES = Previous employment status; NCFM = Number of cohabiting family members; MS = marital status; SRH = self-rated health.

Reference categories: male, unemployed, married, and local residents.
